# Resource redistribution in polydomous ant nest networks: local or global?

**DOI:** 10.1093/beheco/aru108

**Published:** 2014-06-30

**Authors:** Samuel Ellis, Daniel W. Franks, Elva J.H. Robinson

**Affiliations:** ^a^Department of Biology, University of York, York YO10 5DD, UK,; ^b^York Centre for Complex Systems Analysis, University of York, York, YO10 5GE, UK, and; ^c^Department of Computer Science, University of York,York YO10 5GE, UK

**Keywords:** *Formica lugubris*, levels of selection, network analysis, polydomy, self-organization, wood ants.

## Abstract

An important problem facing organisms in a heterogeneous environment is how to redistribute resources to where they are required. This is particularly complex in social insect societies as resources have to be moved both from the environment into the nest and between individuals within the nest. Polydomous ant colonies are split between multiple spatially separated, but socially connected, nests. Whether, and how, resources are redistributed between nests in polydomous colonies is unknown. We analyzed the nest networks of the facultatively polydomous wood ant *Formica lugubris*. Our results indicate that resource redistribution in polydomous *F. lugubris* colonies is organized at the local level between neighboring nests and not at the colony level. We found that internest trails connecting nests that differed more in their amount of foraging were stronger than trails between nests with more equal foraging activity. This indicates that resources are being exchanged directly from nests with a foraging excess to nests that require resources. In contrast, we found no significant relationships between nest properties, such as size and amount of foraging, and network measures such as centrality and connectedness. This indicates an absence of a colony-level resource exchange. This is a clear example of a complex behavior emerging as a result of local interactions between parts of a system.

## Lay Summary

Wood ants nests share resources with neighboring nests, not the whole colony. A single ant colony can either live all in one nest, or split into several separate, but communicating, nests. How and why ant colonies do this is unknown. By treating these separated colonies as networks we show that wood ants exchange food locally, with neighboring nests, without a colony-level plan.

## INTRODUCTION

Resources are usually spread unevenly through the environment, and an important task for many animal species is to redistribute these resources in response to local need. For example, the mammalian body uses the circulatory system to redistribute oxygen through the body, birds may bring food from the environment back to their nest (Krebs et al. 1977), and humans build complex transport networks to move goods to where they are needed (Guimerà et al. 2005). The mechanisms by which these systems function, and how they are organized, profoundly affects their efficiency and robustness to change.

Redistribution of information and resources is particularly challenging for social insects because of the multiple stages through which resources have to be transferred. Food, for example, is often transported by foraging workers from the environment back to the nest, then passed from foraging workers to nest workers, and from these workers to the queens and brood. An additional level of complexity is present if a colony is polydomous. Polydomous colonies are spread between multiple spatially separated nests, socially connected by trails of ants travelling between them (Debout et al. 2007). In a polydomous colony, resources may need to be redistributed between the different nests of the colony, as well as through all the other stages common to social insect colonies. 

Polydomy is a widespread life-history strategy in ants and is thought to have convergently evolved multiple times in a wide variety of ant genera (Debout et al. 2007). However, the mechanism by which the polydomous system functions, and the benefits it provides the colony, remain poorly understood (Debout et al. 2007). Polydomy has the potential to have a profound effect on how a colony relates to the environment (Debout et al. 2007; Van Wilgenburg and Elgar 2007; Cook et al. 2013; Ellis and Robinson 2014). Being distributed through the environment allows a colony to exploit resources, such as food and sunlight, over a larger area. Over a larger area, environmental heterogeneity is likely to mean that, at least at temporarily, some nests will have more of a particular resource than others. Whether resource redistribution occurs, and the mechanism by which it works, is important to understanding how the colony functions.

The redistribution of resources at the global, colony, level has to be mediated by the local interactions between individual nests. The relationship between global and local effects can be investigated using network analysis. Polydomous ant colonies are analogous to networks, with nests as nodes and the trails between nests as connections (Cook et al. 2014). Many tools have been developed to study networks (Newman 2003a; Croft et al. 2008). These tools allow investigation of how local interactions relate to a broader global pattern: in this case, how communicating trails between nests relate to the organization of the polydomous colony.

We used network analysis to investigate how resources are redistributed through polydomous *Formica lugubris* colonies. *Formica lugubris* is a member of the *Formica rufa* species group (sometimes known as the red wood ants) which are the dominant invertebrate predators in woodland across much on Northern Eurasia. They are particularly useful for investigating polydomous nesting because polydomy is flexible both within species and between species (Ellis and Robinson 2014). For example, *F. lugubris* has been reported as monodomous at locations in Finland (Rosengren and Pamilo 1983), Switzerland (Bernasconi et al. 2005), and Ireland (Breen 1979), but polydomous in England (Sudd et al. 1977; Gyllenstrand and Seppä 2003) and at other locations in Switzerland (Bernasconi et al. 2005) and Finland (Rosengren 1971).

Polydomous wood ant colonies form distinct trails of ants travelling between these nests: workers carry food, nesting material, brood, and queens along these trails in both directions (Rosengren and Pamilo 1983). It is unknown how polydomous red wood ants organize the redistribution of resources through the colony. Understanding how these resources are being redistributed through the colony is an important part of understanding the adaptive advantage polydomy may bring the colony. The primary means of resource redistribution through a polydomous colony is likely to be along the trails between the nests. These connections are, therefore, the key to understanding how resources are redistributed between nests. The patterns of connections between nests and how this pattern relates to the properties of the nests themselves will reflect how resources are redistributed through the colony. In this study, we investigate these internest connections. Specifically we consider 2 interlinked questions: 1) How is resource redistribution mediated at the local level between nests? and 2) How do the local interactions relate to the colony-level redistribution of resources?

## METHODS

### Study species and field site

The study was conducted on a large *F. lugubris* population in the Longshaw Estate, Peak District, England (N53° 18′ 33″, E-1° 36′ 9.6″) in July and August 2012. There are no other members of the *F. rufa* group at the site. The 0.95 ha^−1^ site contains a mix of open sparsely planted grassland, deciduous woodland, mixed woodland, and the remains of historic scots pine plantations. A survey over winter and spring 2011–2012 found a total of 921 *F. lugubris* nests on the site (Ellis S, personal observation).

Ants of the *F. rufa* group build distinctive aboveground mounds of pine needles and other leaf litter, over extensive subterranean chambers. These nests can be large, up to a meter in height, and can contain from hundreds to millions of workers (Ellis S, personal observation). If polydomous, a colony will form distinct trails of ants travelling between these nests. Distinctive nests and clear trails are an advantage of using *F. lugubris* as it means that the networks can be readily and reliably mapped.

The location of wood ant nests is likely to be particularly influenced by 2 environmental factors: the location of food in the environment and the temperature of the nest site. A distinctive feature of red wood ant foraging is their reliance on spatially and temporally stable food sources. Red wood ants, along with many other ant species, farm homopterans for honeydew (Hölldobler and Wilson 1990); this actually provides the majority (up to 95%) of the colonies’ nutrient intake (Rosengren and Sundström 1991). For wood ants, this farming is usually of aphid herds in trees (Rosengren and Sundström 1991). In addition to foraging for honeydew in trees, wood ant colonies also hunt and scavenge for arthropods in the canopy, including a large proportion of their protein intake from feeding on the aphids themselves (Cherix 1987; Robinson et al. 2008). The positions of trees in the landscape may influence nest layout not only by affecting the foraging structure but also by shading the nests. Insolation is an important environmental variable for red wood ants (Rosengren et al. 1987; Punttila 1996; Punttila and Kilpeläinen 2009; Sorvari and Hakkarainen 2009). The relationship between insolation and the internal temperature of ant nests is complex, as higher insolation is likely to mean higher temperatures, but also higher variation in temperature (Sorvari and Hakkarainen 2009). Additionally, large wood ant nests can control their internal nest temperature through metabolic heat production, but smaller nests cannot (Rosengren et al. 1987). In general, more insolated, and therefore warmer, nests are likely to have a higher brood development rate (at least in smaller and newly founded nests), but they will be further from trees, which may lower their foraging efficiency.

### Network mapping

We constructed maps of the trail system between and around nests. We were interested in the function of this internest communication. Therefore, for the purpose of this study, a colony is defined by communication (i.e., regular exchange of workers, brood, and other resources) between nests, rather than with reference to intercolony aggression, which has been used in previous studies (e.g., Sorvari and Hakkarainen 2004). Ten polydomous networks were mapped over the site (Table 1). Colonies were chosen for this analysis based on a preliminary colony survey conducted during the early summer. The largest 10 networks from this survey were selected for analysis unless, in the period between the preliminary survey and mapping, they were obscured by the growth of understory vegetation or reduced by destruction of nests in the network, in which case the next largest unmapped colony was used.

**Table 1:** Details of the polydomous networks used in this study (maps; Supplementary Data 1)

Colony	Number of nests	Number of internest trails	Foraged trees	No. of nonforaging nests
1	22	22	38	10
2	10	10	4	6
3	21	30	18	10
4	14	17	4	10
5	14	15	9	6
6	7	6	6	1
7	10	10	14	3
8	9	8	10	1
9	13	13	8	8
10	20	26	7	10

**Figure 1:**
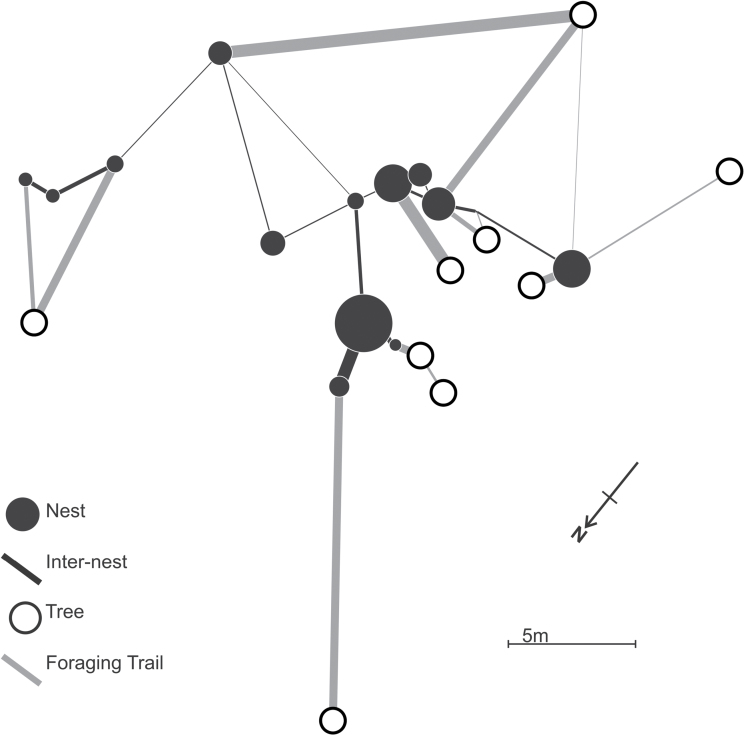
Example of a polydomous network (colony 5; see Table 1) used in this study. Size of a black circle indicates the square root of the nest size and the width of trails indicates their strength. All trees and wood ant nests in the area represented are shown on the map.

All mapping was performed during mid-late summer, when colonies have reached their largest extent (Mabelis 1979), and in warm, sunny conditions to minimize the effect of temperature and weather-based variation in trail activity (Rosengren 1977). The layout of nests, trees, and trails was mapped from the compass bearing of the trails and length of trails measured using a trundle wheel (e.g., Figure 1, further examples in Supplementary Data 1). In addition, we recorded internest trail activity, foraging trail activity, nest population, and canopy cover over each nest. The activity on a trail was measured as distance along a central portion of the trail needed to find 10 ants (in the absence of confounding features such as groups of workers carrying prey). This measure has an advantage over rate-based measures because it is not affected by the speed at which the ants are moving and can be readily converted to the useful measure of number of ants per meter of trail.

The strength of a trail is an important consideration for much of the analysis in the study. How much a trail is used gives an indication of how valuable it is to the nests involved and an estimate of the amount of resource exchange occurring along the trail. Trail strength (*S*) is estimated as the total number of ants travelling along a trail between nests a and b taking into account the size of the nests at each end of the trail. Multiplying the number of ants per meter (*w*) by the length of the trail (*l*) gives an estimate of the amount of resource exchange (or at least the potential amount) occurring between nests but does not give a good impression of the value of the trail to the nests because it does not take into account the sizes of the nests at each end of the trail. The sizes of the connected nests will strongly affect the number of workers available to travel along the trail, masking the relative value that trail to the nests as a channel for resource exchange. We account for this by dividing the total number of workers on the trail by the mean population of the nests (see below) the trail is connecting (*p*_a,b_). The calculation of the strength of the trail between nests a and b is shown in Equation 1.

$$S_{\text{a},\text{b}}=\frac{wl}{p_{\text{a},\text{b}}}$$ (1)

The worker population of wood ant nests can be accurately estimated using a mark–release–recapture method based on marking after surface disturbance (Chen and Robinson 2013); however, it is time consuming and disruptive to the nests. We used the mark–release–recapture method to calibrate a quicker, but less accurate, estimate of nest population calculated from nest-mound volume (Chen and Robinson 2013). We measured the volume of all the nests as half the volume of an ellipsoid based on measurement of 2 perpendicular diameters and nest height (Chen and Robinson 2013). One nest per colony was randomly chosen to calibrate volume measurement with a mark–release–recapture estimate of nest population. For greater reliability, these calibration measurements were pooled with equivalent data from a separate study (Ellis S, unpublished data) using smaller networks at the same site (*n* = 15). We fitted a linear regression to give a site-specific relationship between nest volume and estimated nest population (linear regression: *R* ^2^ = 52.7, df = 1,24, *P* < 0.001). We used the values of the regression to give estimates of the nest population of each nest. To avoid ambiguity, we used nest size to refer to the population size of a nest, rather than its physical size.

Distinct trails of ants form between nests and trees with aphid herds. The majority of the ants in these trails are likely to be foragers, collecting honeydew from the aphids and then returning with it to the nests (Gordon et al. 1992). The number of ants from a nest visiting a foraging tree is, therefore, a measure of the amount of foraging (or potential amount of foraging) being performed by a nest. We define a foraging trail as a clear trail (more than 10 ants in 40cm) from a nest to a tree. The number of ants on a foraging trail was measured in the same way as for the internest trails. Foraging trail strength was calculated as the number of ants on the trail divided by the population of the nest the trail originates from; this is to control for the internal demand of the foraging nest. The amount of foraging performed from a nest was calculated by summing the strengths of all of the foraging trails connected to a nest. This measure only uses the foraging trails to trees and does not take into account any foraging being performed elsewhere, for example, in the leaf litter. However, it is likely that the proportion of nutriment provided by the aphid herds is very high (up to 95% has been suggested: Rosengren and Sundström 1991) as they are a source of both honeydew and protein for the colonies (Cherix 1980, 1987; Robinson et al. 2008). Using the strength of the trails to trees as a measure of amount of foraging will take into account the majority of the food that a nest collects. Nests are considered “nonforaging” if they do not form trails to any trees; this does not necessarily mean that the nests perform no foraging at all, simply that they do not form foraging trails directly to aphid-bearing trees.

The amount of foraging performed by a nest can be used to calculate the foraging differential of an internest trail. The foraging differential is the difference in amount of foraging performed by the nests connected by a trail. In analysis of foraging differentials, trails between 2 nonforaging nests are excluded because the foraging differential is always 0 and is therefore unsuitable for analysis.

The amount of insolation received by a nest is largely determined by the canopy cover over the nests. Canopy cover over nests was estimated using digital photographs taken vertically 30cm above the highest point of the nest. ImageJ (Rasband 2012) was then used to count the number of dark pixels (black/white intensity threshold = 255) in the 8-bit version of the image to give the percentage canopy cover (for a similar method, see Robinson et al. 2008).

### Network analysis

This study investigates the relationship between the nest and trail properties and network structural properties. All network analysis was performed in R (R Development Core Team 2011), using the igraph (Csardi and Nepusz 2006) and nortest packages (Gross and Ligges 2012). Three main nest-level network parameters were measured for the analysis: connectedness, centrality, and assortativity. These measures allow us to ask biologically meaningful questions about the position of the nests in the network.

Connectedness is a measure of how much resource exchange a nest is facilitating. It can be measured simply as degree, which is the number of other nests connected to the nest. It can also be calculated as weighted degree, which is the sum of the strength connections to the other nests (Croft et al. 2008). We use both measures.

Centrality is a measure of the extent to which a nest occupies an important position in the network (Newman 2003b). We use 2 network metrics, node betweeness and closeness, to estimate the centrality of a nest to the network. Node betweeness measures the amount of information flow through a node and is measured as the total number of shortest paths between all pairs of nests in the network that pass through the nest. If ants were travelling freely and optimally through the network, nests with the highest node betweeness would be passed through most often. It was calculated both by considering all trails as equal strength (unweighted node betweeness) and by taking into account the strengths of the trails when calculating the shortest path (weighted node betweeness). Closeness is a measure of how many trails must be passed along from a nest to reach all other nests in the network. So ants starting from a nest with high closeness can reach all other nests in the network by travelling along fewest trails. This was calculated as both a simple count and weighted by trail strength.

Trail betweeness is a measure of optimal flow through a particular trail in the network. As node betweeness measures the number of shortest paths passing through a node, so trail betweeness measures the number of shortest paths passing through a particular trail. We also calculated trail betweeness and weighted trail betweeness for the internest trails.

Assortativity measures the extent to which nests with a particular property are connected in the network (Newman 2003b). We calculated both unweighted and weighted (by trail strength) network associations using Newman’s assortativity coefficient *r* (Newman 2003a). We examined the assortativity of nest size, amount of foraging, and weighted degree (called degree correlation) within the networks.

To account for autocorrelations, we used a null model based on 1000 node-label permutations using the quadratic assignment procedure (QAP). This preserves the network structures while nest or trail properties are randomized (Croft et al. 2011). Where analysis is performed on pooled data from all the colonies, randomizations were constrained to within each colony. All significance values based on network measures were calculated using QAP. All analyses not based on QAP fit the assumptions of the statistical test used. All quoted values are mean ± standard error.

## RESULTS

### Local structure

**Figure 2:**
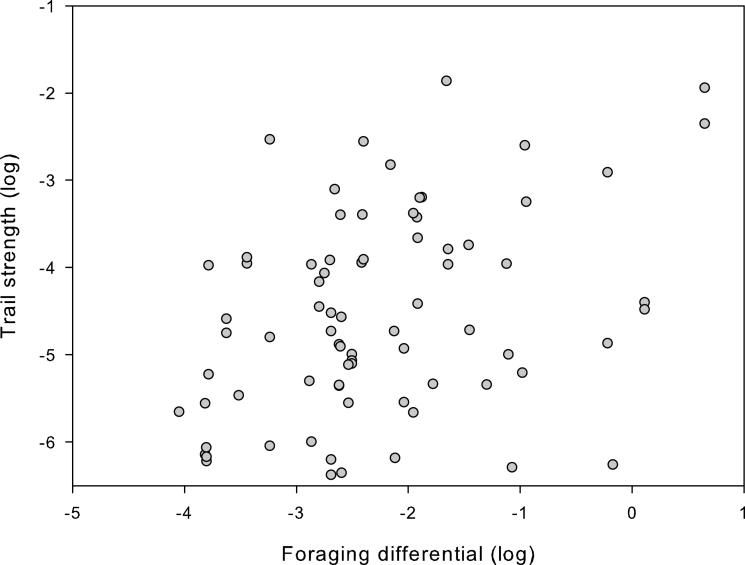
Relationship between foraging differential and trail strength. There is a weak but significant positive correlation between the variables (Spearman: ρ = 0.36, *n* = 79, *P* = 0.015). Axes are logged for presentation due to the large range of values of both foraging differential and trail strength.

Our results clearly show that strength of an internest trail is related to the foraging properties of the nests that it connects rather than being related to any colony-level network properties. The strength of internest trails gives an indication of how resource exchange is facilitated at a local level, between individual nests. Trail strength is a measure of the investment a nest puts into the connection to another nest. Analysis of trail strengths is, therefore, representative of the value a nest places on a particular trail, which gives insights into how the trails are being used. By examination of the network maps, internest trails can be split into 3 categories: those between 2 foraging nests (F-F; 28% of trails), those between a nonforaging and a foraging nest (nF-F; 50% of trails), and those between 2 nonforaging nests (nF-nF; 22% of trails). There is no significant relationship between the type of trail and the strength of a trail (Anova, *F* = 1.13, *n* = 177, *P* = 0.664). However, there is a significant positive correlation between the foraging differential (the difference in amount of foraging performed at nests at each end of the trail) and the strength of the trail on nF-F trails (Pearson: *r* = 0.36, *n* = 79, *P* = 0.019; Figure 2). There is no significant correlation between foraging differential and trail strength on F-F trails (Pearson: *r* = 0.04, *n* = 44, *P* = 0.464). If the data from nF-F trails and F-F trails are combined, there is no significant relationship between foraging differential and trail strength (Pearson: *r* = 0.2, *n* = 123, *P* = 0.126).

The length of a trail is likely to be important for ants travelling between nests. Overall, F-F trails are significantly longer than other types of trail (F-F: 6.72±1.33 m, nF-F: 3.18±0.24 m, nF-nF: 2.61±0.40 m; Anova: *F* = 7.80, *n* = 177, *P* = 0.001). For nF-F trails, longer trails are both significantly stronger (Pearson: *r* = 0.30, *n* = 77, *P* = 0.013) and have a higher foraging differential (Pearson: *r* = 0.12, *n* = 79, *P* = 0.042) than shorter trails. However, F-F trails show no significant relationship between trail length and either trail strength (Pearson: *r* = 0.32, *n* = 44, *P* = 0.26) or foraging differential (Pearson: *r* = 0.04, *n* = 44, *P* = 0.075).

There is evidence of significant positive assortment by weighted degree at least within some networks (Table 2). This is probably in part a consequence of the relationship between foraging differential and trail strengths. Assortment by weighted degree is indicative of clusters of high resource exchange within the network.

**Table 2:** Weighted degree correlation of the polydomous *Formica lugubris* colonies

Colony	*r*	*P*
1	0.57	<0.001*
3	0.28	0.231
4	0.43	0.003*
5	0.20	0.282
6	0.53	0.024*
7	0.25	0.234
8	0.67	0.018*
9	0.62	0.063**
10	0.13	0.627
11	0.46	0.021*

*r* is Newman’s assortativity coefficient; a positive value shows positive assortment.

*P < 0.05; **P < 0.1. All *P*-values have been adjusted with a Bonferroni correction to control for repeated assortativity tests on the same colony (Supplementary Data 2).

### Colony-level structure

We investigated the relationship between the colony network structure and properties of the nests that make up the colony by examining correlations between network structure variables (connectedness and centrality) with nest properties (size, canopy cover, and amount of foraging). We found no significant relationship between the network structure and any of the nest variables (Supplementary Data 2). Similarly, there is no significant association by either size or amount of foraging (Supplementary Data 3).

The number of nests in a colony might be expected to be linked to environmental and internal colony variables. However, there is no significant relationship between the number of nests and the mean canopy cover over the nests of the colony (Spearman: ρ = 122, *n* = 10, *P* = 0.48). Similarly, there is no significant relationship between the number of nests in the colony and the size of the nests in the network (Pearson: *r* = 0.31, df = 8, *P* = 0.76). It was not necessary to use QAP for these nest number statistics as they are not network related.

The strength of a trail is a measure of actual flow of ants within the polydomous network; it might, therefore, be expected to relate to the trail betweeness, which is a measure of optimal flow through the network. However, there is no significant relationship between trail strength and trail betweeness in any of the networks (Supplementary Data 4). Similarly, there is no significant relationship between the type of trail and either trail betweeness or weighted trail betweeness in any of the networks (Supplementary Data 5).

More restricted flow of workers through the network could occur if workers from a particular nest use the foraging trails from neighboring nests. In the case of nF-F trails, workers from the nF nest could use the foraging trails from the F nest; this would increase the amount of foraging the F nest is carrying out, relative to its size. The number of extra foragers should scale with the size of the nF nest, resulting in a relationship between the size of the nF nest on the trail and the relative amount of foraging from the F nest. However, there is no significant relationship between the size of the nF nests and the relative amount of foraging occurring from the F nest on nF-F trails (Pearson: *r* = 0.06, *n* = 79, *P* = 0.24). This suggests that moving from internest trails to foraging trails is unlikely to play a significant role in resource redistribution, at least on nF-F trails.

### Relationship between nest variables

**Figure 3:**
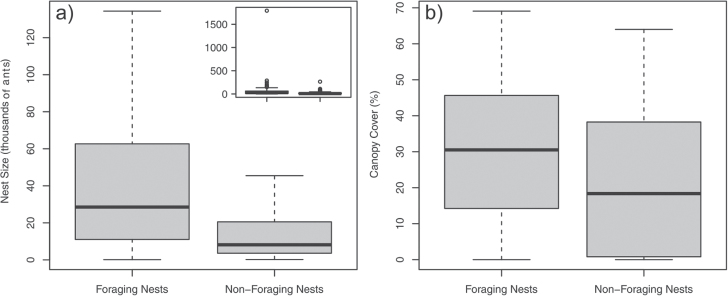
Summary of relationships between nest variables, in both figures *n* = 139. (a) Foraging and nest size, without outliers (inset with outliers). (b) Foraging and canopy cover.

Nest size, canopy cover over the nest (as a proxy for insolation), and amount of foraging are ecologically important nest traits. The relationships between these variables were analyzed within the context of the network. The results suggest that the most important variable is the difference between a nest foraging or not foraging. Foraging nests are larger (F:72630±23900 workers vs. nF:22760±4923 workers) and in darker areas (F:30±2.6% canopy cover vs. nF:21±2.4% canopy cover), whereas nonforaging nests are smaller and in lighter areas (foraging and nest size: Anova, *F* = 7.09, *n* = 139, *P* = 0.001; foraging and canopy cover: Anova, *F* = −3.5, *n* = 139, *P* = 0.003; Figure 3). There is no significant relationship between the canopy cover and size of a nest (Pearson: *r* = 0.12, *n* = 139, *P* = 0.084). Larger foraging nests do not forage proportionally less than smaller foraging nests (Pearson: *r* = −0.08, *n* = 76, *P* = 0.233). Similarly, foraging nests show no significant relationship between amount of foraging and canopy cover (Pearson: *r* = −0.01, *n* = 76, *P* = 0.613).

## DISCUSSION

Our study shows that *F. lugubris* polydomous nest networks are structured around exchange of foraged resources between pairs of nests, rather than at the level of the colony. This is evident in the positive relationship between internest trail strength and foraging differential and the absence of a relationship between trail betweeness (a measure of optimal movement through a network) and trail strength. Both results suggest that individual ants are not moving through the whole network to redistribute resources but rather travelling only locally to nests to which they are directly connected. This is supported by the relationships between trail length and the other trail properties. In a colony based around local resource exchange, it would be worthwhile to construct long trails between distant nests only if there is an important gain to be made from the connection. This is what we found in the *F. lugubris* networks. In this case, the gain is probably resource exchange, as this pattern is only observed in the nF-F trails. There is no evidence that workers from nonforaging nests use the foraging trails of their neighbors, suggesting that resource exchange is occurring at the nest, rather than on the foraging trails from the nest. Further study is needed to establish the mechanism of this resource exchange, and how it relates to the movement of individual workers.

If the network is structured around colony-level resource exchange, a correlation would be expected between nest properties (such as size or amount of foraging) and network variables (such as centrality and connectedness). For example, in a colony optimized to redistribute foraged resources, foraging nests might be expected to be well connected because they are acting as a hub from which other nests collect resources. Or it might be expected that nonforaging nests show higher centrality, as they are acting as a link between separate foraging patches and maintaining colony cohesion. Our finding that there is no relationship between any of these variables suggests a lack of colony-level organization of resource redistribution in polydomous *F. lugubris* colonies.

The lack of colony-level organization is further highlighted by the lack of relationship between the number of nests in a colony and either canopy cover or sizes of nests in the network. It might be expected there is an optimum number of nests for a colony dependent on external (insolation) or internal (size) conditions. The absence of relationship between degree of polydomy and canopy cover is interesting as it is inconsistent with previous work on wood ants, which has suggested a link between polydomy and insolation (Sudd et al. 1977; Sorvari and Hakkarainen 2005). Indeed, it has been argued that a higher degree of polydomy is important to survival in deeper woodland (Punttila 1996). However, the difference in findings between the studies may be caused by difference in the habitats. For example, in contrast to many previous studies (e.g., Punttila 1996), this study was undertaken in the absence of any other members of the *F. rufa* group. Further investigation is needed to establish if this lack of relationship between canopy cover and degree of polydomy is just a local pattern or a more general feature of wood ant ecology.

The concept of a network built around local interactions shares features with other aspects of wood ant life history. Previous studies of monodomous colonies have found that foragers display a high degree of site allegiance and route fidelity (Rosengren 1971; Rosengren and Fortelius 1986; Gordon et al. 1992). Polydomy in *F. lugubris* could function by a similar mechanism based on workers showing loyalty to a particular nest and providing food for, or taking food from, neighboring nests. This mechanism would result in the observed pattern of higher numbers of workers visiting (or visiting from) nests with a foraging excess. A particularly clear pattern would be expected between foraging and nonforaging nests, as the nonforaging nests have no other substantial source of food. This pattern is what we found in the *F. lugubris* polydomous networks.

Mechanisms similar to those implied by our results have been used in theoretical models of polydomy (Schmolke 2009; Cook et al. 2013). In these models, workers are loyal to a particular nest and treat other nests of the colony as food sources. This mechanism would create a network based on the interactions between partly autonomous nests rather than a colony-level organization. A related mechanism has been observed in other ant species based on a transporter class specializing in carrying resources along internest trails (Dahbi et al. 1997; Pfeiffer and Linsenmair 1998). Further investigation is necessary to distinguish between these mechanisms in *F. lugubris*.

Route fidelity is a feature of foraging in many ant species, particularly species that rely heavily on honeydew for nutrition (Rosengren 1977; Tilles and Wood 1986; McIver 1991; Quinet and Pasteels 1996; Gordon 2012). The wide phylogenetic distribution of this mechanism may suggest that trail fidelity is an efficient way to forage for spatially and temporally stable food sources. For nests in a polydomous colony, other nests in the network may act as spatially and temporally stable food sources, which would make it beneficial to exploit them using a mechanism similar to that used to exploit stable food sources. Resource redistribution in polydomous ant colonies may, therefore, be an example of the adaption of existing behaviors to new tasks: in this case, foraging behaviors to being used to facilitate resource exchange.

The lack of colony-level organization suggests a certain level of autonomy for nests within the network. This nest autonomy also has the potential to facilitate division of labor between nests in the network. Similarly to within the colony itself, where workers often specialize at different tasks (e.g., foraging, brood care), nests within a colony may specialize at, for example, foraging or brood production. Division of labor may explain the presence of so many nonforaging nests in our *F. lugubris* polydomous networks. For example, the smaller and better insolated nonforaging nests are likely to have a internal temperature different from that of the larger, shaded foraging nests, perhaps providing a better temperature for brood development. Similarly, nonforaging nests could be important for collection of other resources that the colony needs such as nesting material. It is also important to note that our definition of nonforaging nest does not necessarily mean that a nest is not foraging at all, just that it is not forming foraging trails to trees. It may be that smaller nonforaging nests are actually playing an important role as bases for scavenging and hunting arthropod prey. This contrasts with studies of polydomy in some other ant species that have been observed to build smaller nests, without brood, near to honeydew sources as temporary bases for foragers (McIver and Steen 1994; Lanan et al. 2011; Csata et al. 2012; Lanan and Bronstein 2013). In these colonies, there is a clear division of labor between the foraging bases and the permanent, brood-rearing, nests. Further investigation is needed to establish the extent and role of division of labor in polydomous colonies.

The concept of “nest traits” as opposed to “colony traits” raises interesting questions about levels of selection in this species. The level at which selection acts is an important question in the study of evolution. In social insects, the problem becomes even more complex by the addition of colony-level selection, as well as selection on the individual, and ultimately the gene (Bourke and Franks 1995). Polydomous colonies have the potential for yet another level of selection: the nest (Banschbach and Herbers 1996; Debout et al. 2007). In this system, it certainly seems like there is the potential for nest-level selection. Nests in the *F. lugubris* network seem to show a certain degree of autonomy: at least in terms of acquisition of resources, each nest appears to be acting either independently or only with neighbors. This raises the intriguing possibility of nests that are better at collecting resources than others. This might result in increased production of brood by some nests, which (depending on the levels of brood and queen exchange between nests) may result in a selective advantage to gathering resources at the expense of the rest of the colony. This may be manifested in the nonforaging nests found in the *F. lugubris* polydomous networks. Rather than providing an adaptive benefit to the colony, the nonforaging nests could be parasitic on the effort of the foraging effort of the rest of the colony, that is, nonforaging nests are a cheating strategy in polydomous colonies. The nonforaging nests may be smaller simply because they are completely reliant on other nests for resources, and perhaps this strategy may only be possible if the nest has a small population. However, further study, especially of the level of brood and queen exchange between nests, is needed to establish if the conditions for nest-level selection are being met by polydomous colonies.

Some studies of polydomous networks have found evidence of efficient and robust nest network organization at the colony level (e.g., Aron et al. 1990; Latty et al. 2011). Analysis of the polydomous networks of a variety of ant species (including *F. lugubris*) has suggested that the networks are locally and globally efficient for resource transportation (Cook et al. 2014). One of the characteristics of locally and globally efficient networks is the pattern of many local connections with a few longer connections (Watts and Strogatz 1998). The longer trails may represent an adaptation to increase the robustness of the entire nest network: this is indicative of a higher, colony-level organization of polydomy (Cook et al. 2014). In the current study, there is no relationship between the strength and length of trails between pairs of foraging nests; these longer trails may be the trails playing an important role in maintaining colony cohesion and adding a measure of robustness to the networks. Longer connections that increase network efficiency and robustness have been found in other systems including termite nest galleries (Perna et al. 2008), bottlenose dolphin social networks (Lusseau 2003), and Trinidadian guppy social systems (Croft et al. 2004). In these examples, the relationship between local connections and the global organization has significant implications for the structure of the communities. In wood ant polydomous networks, the link between the local internest interactions and colony-level social organization has comparably significant implications for how the colony functions and how the colony reacts to changes in the environment, which makes it an important area for further investigation.

Local interactions that build up to more complex, colony-level behaviors are a recurring theme in the study of social insects. The raiding patterns of army ants (Franks et al. 1991), house-hunting in *Temnothorax albipennis* (Robinson et al. 2011), and honey bee comb formation (Camazine 1991), to name only a few, have all been shown to be driven by the interactions in behavior of individuals, rather than by any central control or planning. This is not limited to social insects and has been found in many other biological systems (e.g., vertebrate movement: Couzin and Krause 2003; human decision-making: Krause et al. 2010; plant growth: Leyser 2011). This self-organized pattern appears to be reflected in the polydomous nesting strategy of *F. lugubris* as the behavior seems to be mediated by the local interactions between individual nests, with no central organization and limited colony-level structure.

## SUPPLEMENTARY MATERIAL

Supplementary material can be found at http://www.beheco.oxfordjournals.org/

## FUNDING

This work was supported by the National Environmental Research Council (S.E.), the National Trust (S.E.), and the Royal Society (E.J.H.R.).

## Supplementary Material

Supplementary Data
