# Supplementary material for: Resource redistribution in polydomous ant nest networks: local or global?
Source: Behav Ecol. 2014 Jun 30;25(5):1183–91. doi: 10.1093/beheco/aru108 (PMC4160112; doi:10.1093/beheco/aru108)
Supplement: Supplementary Data [file supp_25_5_1183__index.html]

Resource redistribution in polydomous ant nest networks: local or global? — Resource redistribution in polydomous ant nest networks: local or global? — Supplementary Data 

# Resource redistribution in polydomous ant nest networks: local or global?

## Supplementary Data

Data files

**Files in this Data Supplement:**

- Supplementary Data - Supplementary Data
- Supplementary Data - Supplementary Data
- Supplementary Data - Supplementary Data
- Supplementary Data - Supplementary Data
- Supplementary Data - Supplementary Data
