# Supplementary material for: Resource redistribution in polydomous ant nest networks: local or global?
Source: Behav Ecol. 2014 Jun 30;25(5):1183–91. doi: 10.1093/beheco/aru108 (PMC4160112; doi:10.1093/beheco/aru108)
Supplement: Supplementary Data [file supp_aru108_Supplementary_Data_5.pdf]

Supplementary Data 5: Differences in trail betweeness for different types of trail for weighted (F-F, F-nF, nF-nF) and unweighted networks. All statistics are Kruskall-Wallis tests with a p value constructed form constrained randomisation (see methods). If nest number was less than 10 the statistics were considered unreliable and not included.

**Colony**	**Unweighted**		**Weighted**	
(n)	*Kruskall -Wallis* χ^2	*p*	*Kruskall -Wallis* χ^2	*p*
**1** (22)	2.64	0.282	1.95	0.416
**2** (10)	1.99	0.552	1.18	0.674
**3** (21)	0.57	0.755	1.19	0.564
**4** (14)	2.69	0.258	4.67	0.069
**5** (14)	1.44	0.541	1.00	0.631
**6** (7)	-	-	-	-
**7** (10)	0.01	0.844	0.01	0.838
**8** (9)	-	-	-	-
**9** (13)	2.39	0.309	1.89	0.480
**10** (20)	3.39	0.148	0.25	0.893
