# Supplementary material for: Resource redistribution in polydomous ant nest networks: local or global?
Source: Behav Ecol. 2014 Jun 30;25(5):1183–91. doi: 10.1093/beheco/aru108 (PMC4160112; doi:10.1093/beheco/aru108)
Supplement: Supplementary Data [file supp_aru108_Supplementary_Data_3.pdf]

Supplementary Data 3: Assortativity of nest size and amount of foraging within polydomous *F. lugubris* colonies, *r* is Newman's Assortativity coefficient; a positive value shows positive assortment. An asterisk indicates p<0.05 and a dot indicates p<0.1) All p-values have been adjusted with a Bonferroni correction to control for repeated assortitivity tests on the same colony (table 2)

**Colony**	**Size**		**Amount of Foraging**	
	r	*p*	*r*	*p*
**1**	-0.05	1.00	-0.10	1.00
**2**	-0.42	0.273	-0.49	0.115
**3**	0.00	1.00	-0.12	0.993
**4**	-0.37	0.039*	-0.04	1.00
**5**	-0.32	0.252	-0.18	1.00
**6**	0.28	0.225	-0.09	1.00
**7**	0.18	0.249	0.51	0.033
**8**	-0.05	1.00	0.07	0.477
**9**	-0.25	0.798	-0.12	1.00
**10**	0.10	0.234	0.15	0.345
