# Supplementary material for: Resource redistribution in polydomous ant nest networks: local or global?
Source: Behav Ecol. 2014 Jun 30;25(5):1183–91. doi: 10.1093/beheco/aru108 (PMC4160112; doi:10.1093/beheco/aru108)
Supplement: Supplementary Data [file supp_aru108_Supplementary_Data_1.pdf]

Supplementary Data 1: Network maps of the colonies used in this study. All circles are nests, and lines between circles are internest trails. Closed circles are foraging nests with visible trails to trees. Nests represented as grey circles have no trails to trees and are therefore classified as non-foraging.

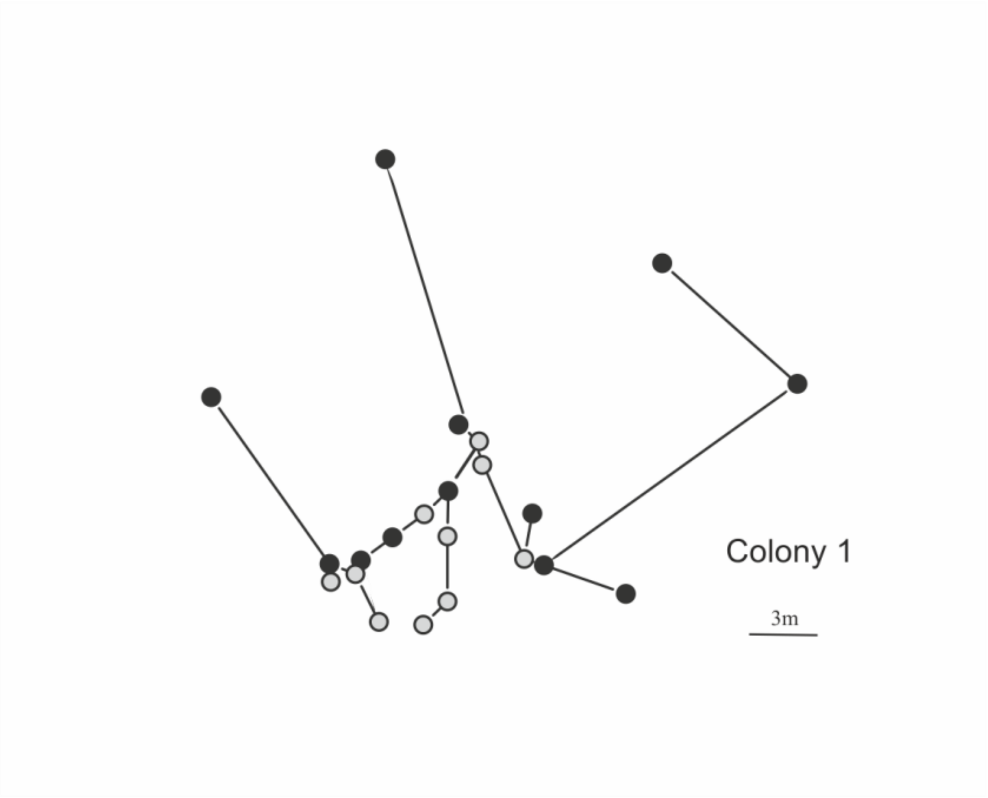

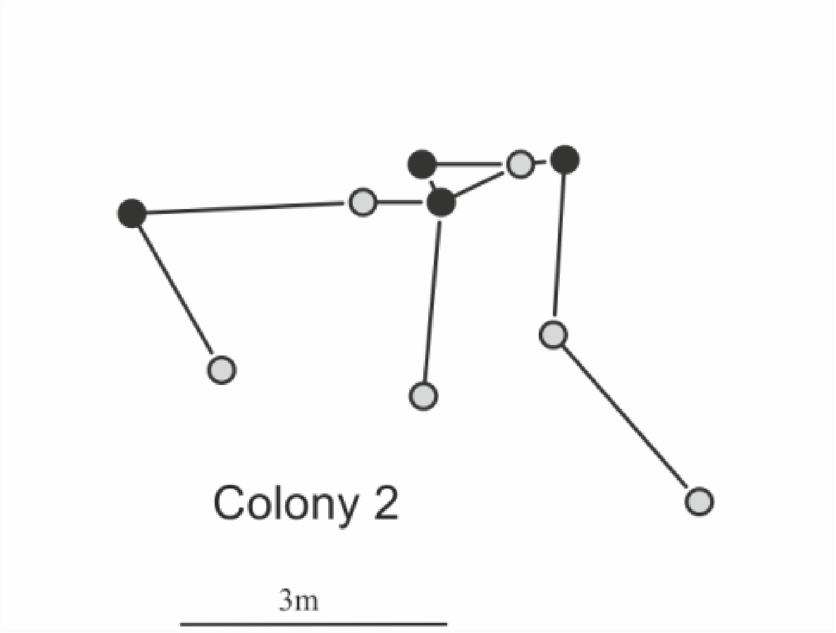

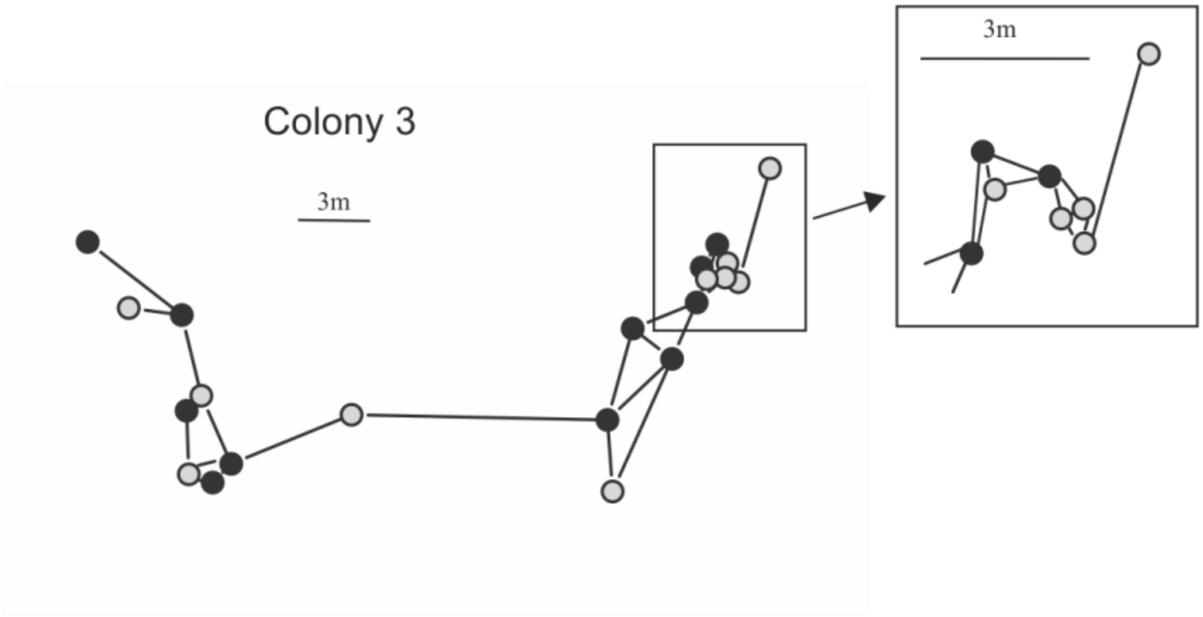
Colony 3
3m
3m

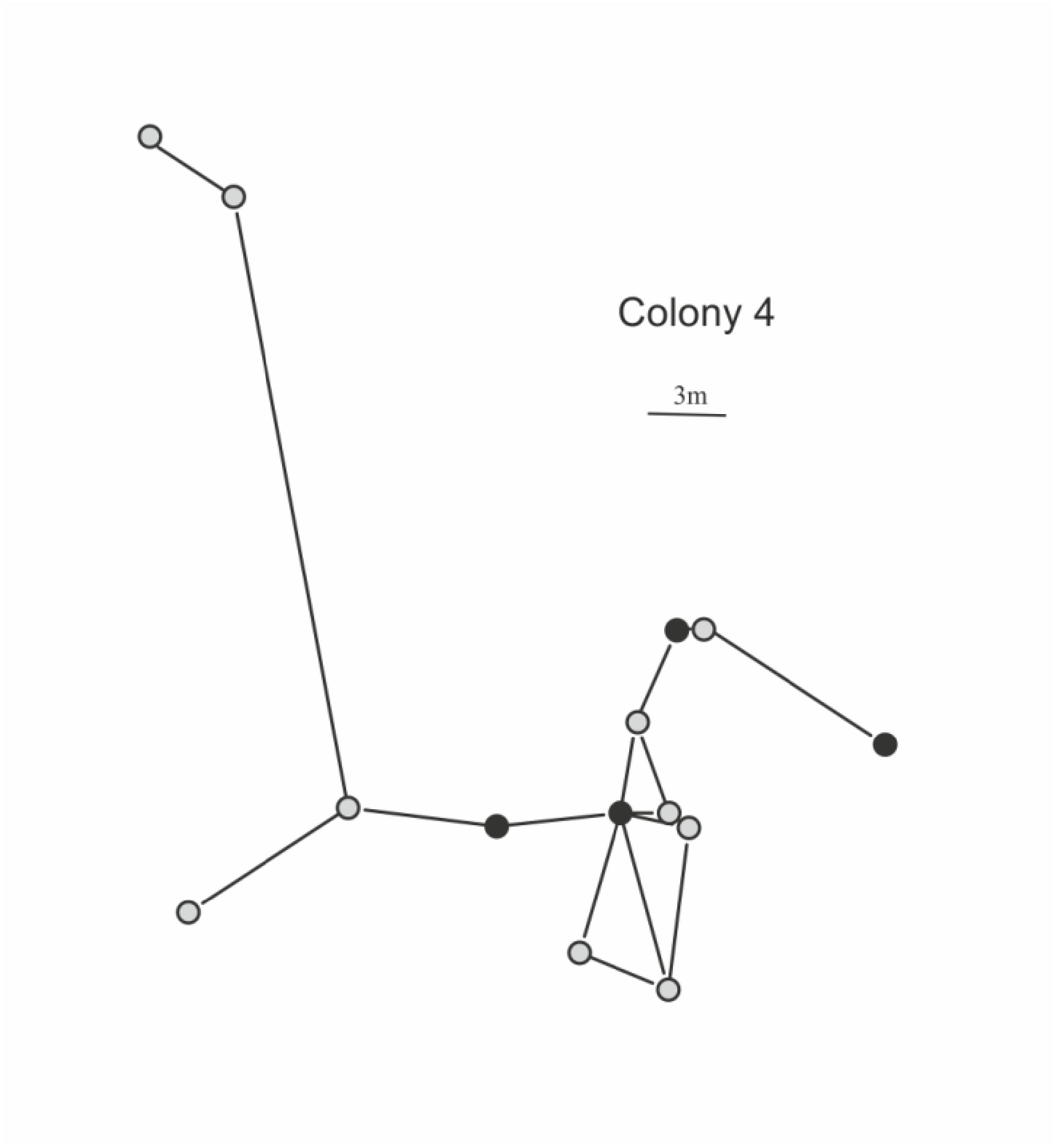
Colony 4
3m

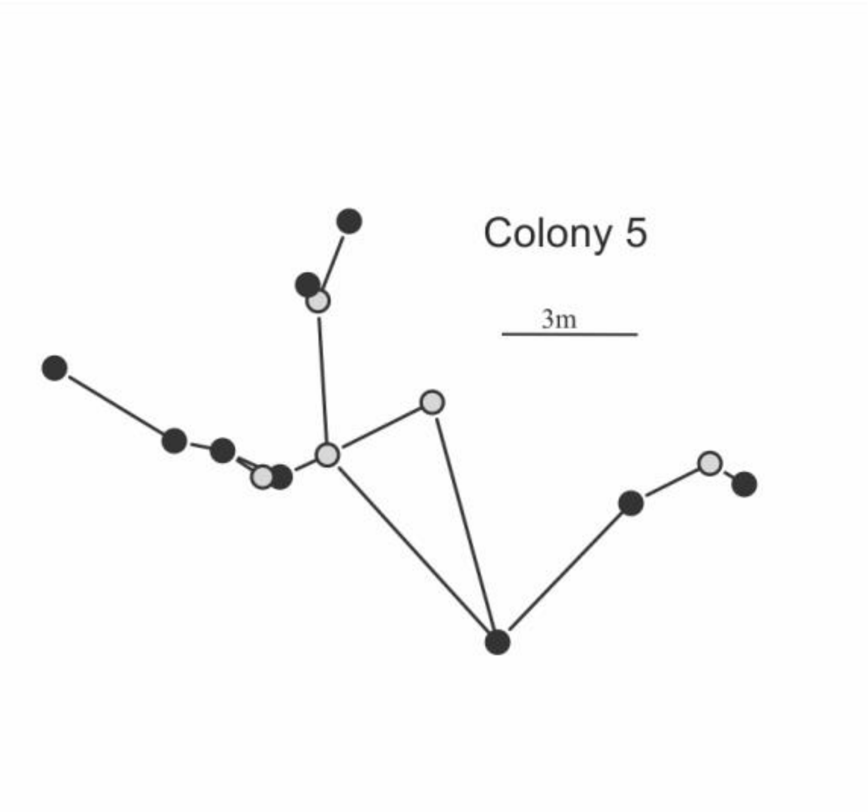
Colony 5
3m

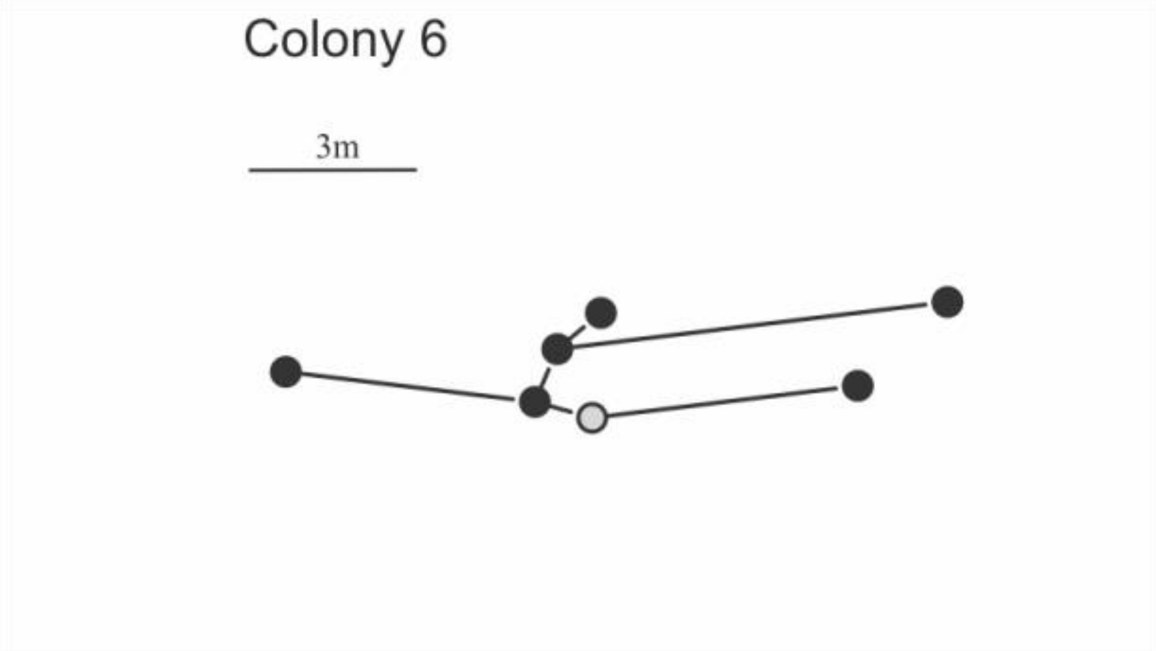
Colony 6
3m

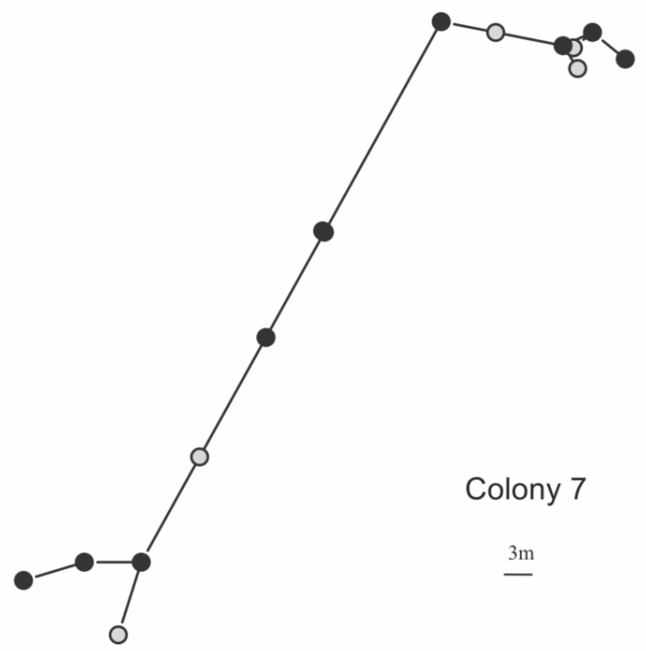
Colony 7
3m

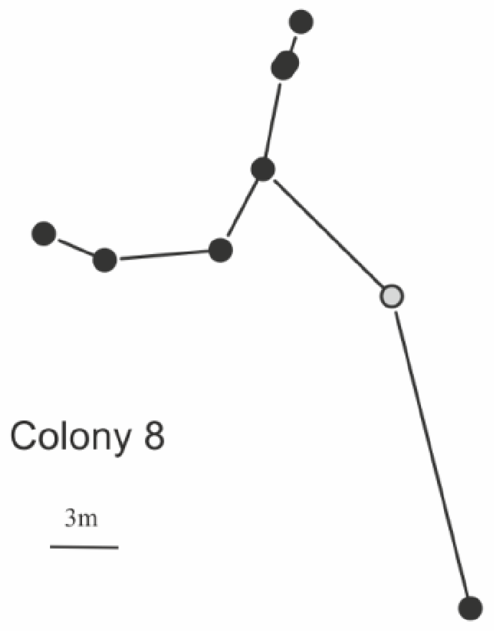
Colony 8
3m

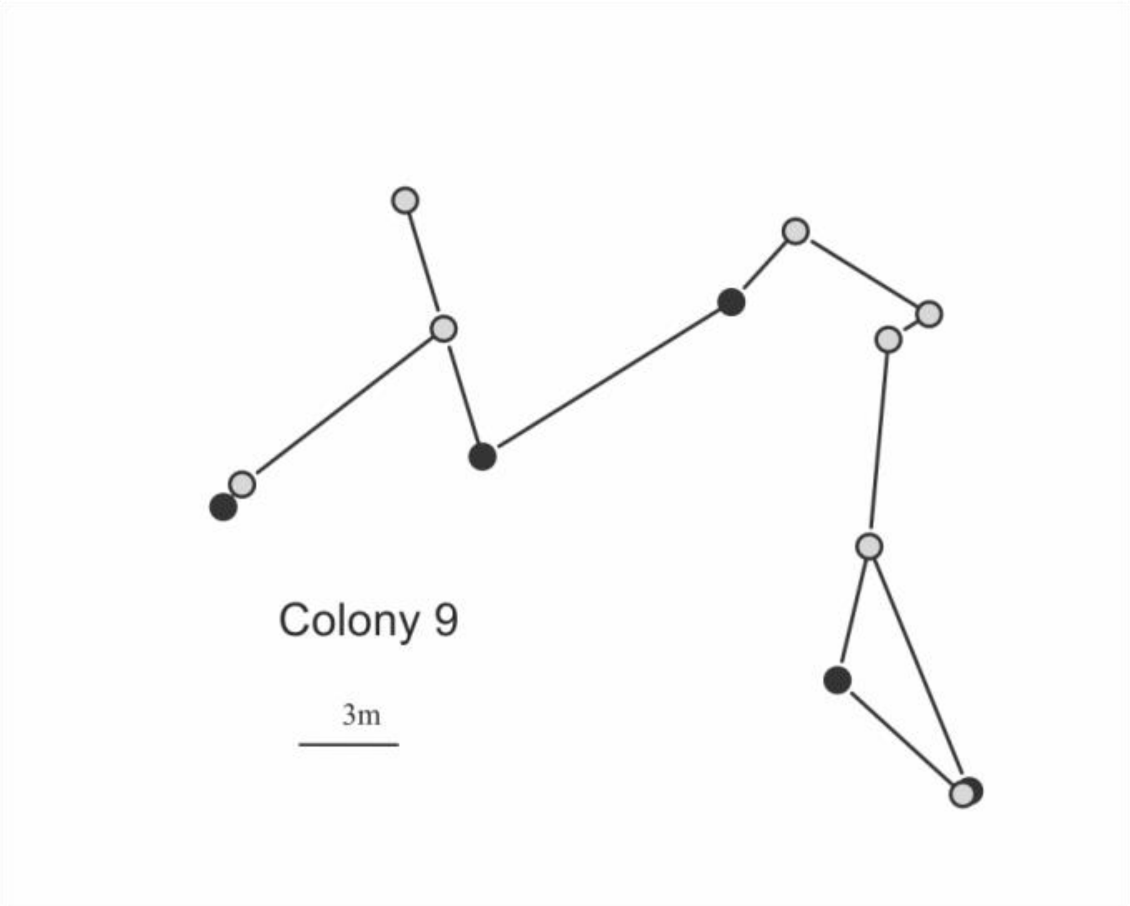
Colony 9
3m

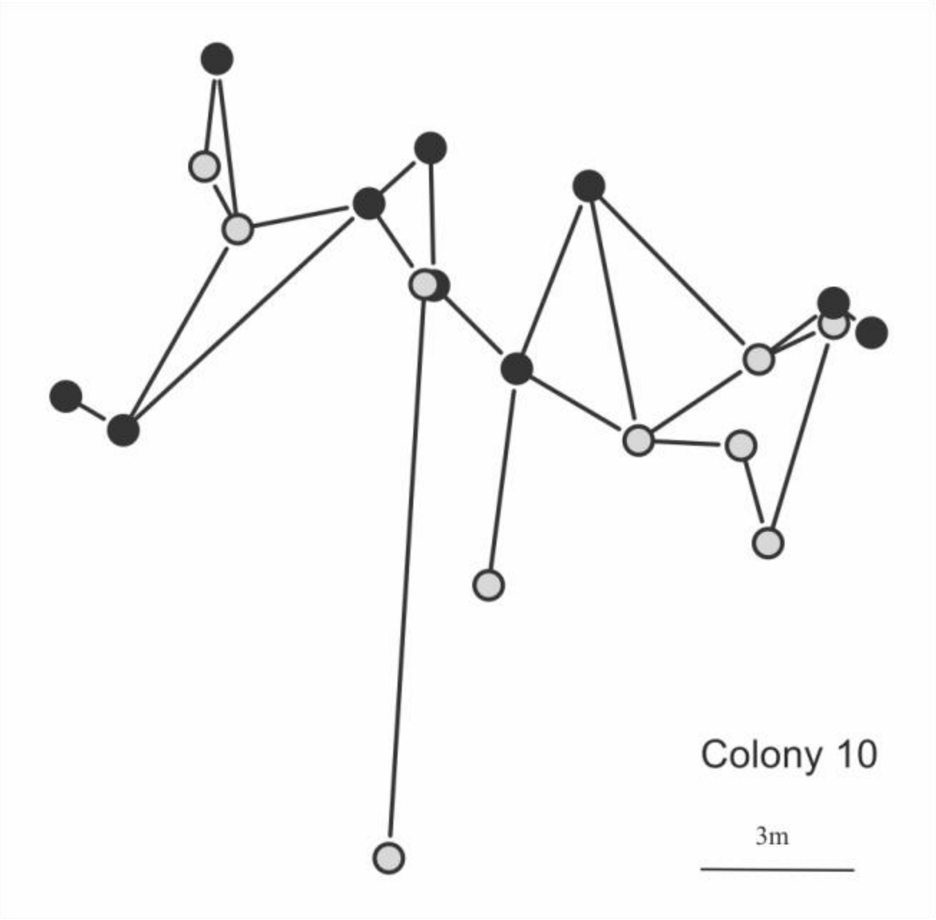
Colony 10
3m
