# Supplementary material for: Resource redistribution in polydomous ant nest networks: local or global?
Source: Behav Ecol. 2014 Jun 30;25(5):1183–91. doi: 10.1093/beheco/aru108 (PMC4160112; doi:10.1093/beheco/aru108)
Supplement: Supplementary Data [file supp_aru108_Supplementary_Data_2.pdf]

Supplementary Data 2. Summary of tests of the relationships between nest properties (rows) and network structural properties (columns) in *F. lugubris* polydomous colonies. ρ values refer to the Spearman's rank correlation coefficient, χ^2 to Kruskall Wallis statistic. Centrality tests were performed individually for each network as the values are reliant on the number of nodes and therefore cannot be directly compared. If n<10 statistics were considered unreliable and are not included. We performed the Bonferroni correction to control for repeated testing on the same data.

	**Connectedness**	**Centrality**	
	Degree (n=140)	Closeness	Betweeness
**Size**	*Unweighted*<br>(χ^2=8.45,p=0.952)<br>*Weighted*<br>(ρ =-208,p=0.152)	*Unweighted*<br>p<0.05: 0/10 Networks<br>*Weighted*<br>p<0.05: 1/10 Networks	*Unweighted*<br>p<0.05: 1/10 Networks<br>*Weighted*<br>p<0.05:0/10 Networks
**Foraging vs. Non-Foraging**	*Unweighted*<br>(χ^2=0.921,p=1.000)<br>*Weighted*<br>(χ^2=0.214,p=1.000)	*Unweighted*<br>p<0.05: 0/10 Networks<br>*Weighted*<br>p<0.05: 0/7 Networks	*Unweighted*<br>p<0.05:0/10 Networks<br>*Weighted*<br>p<0.05:0/7 Networks
**Amount of Foraging**	*Unweighted*<br>(χ^2=4.14,p=1.000)<br>*Weighted*<br>(ρ =0.14,p=0.856)	*Unweighted*<br>p<0.05: 0/10 Networks<br>*Weighted*<br>p<0.05:0/7 Networks	*Unweighted*<br>p<0.05: 0/10 Networks<br>*Weighted*<br>p<0.05: 0/7 Networks
**Canopy cover**	*Unweighted*<br>(χ^2=1.06,p=1.000)<br>*Weighted*<br>(ρ =0.02,p=1.000)	*Unweighted*<br>p<0.05: 0/10 Networks<br>*Weighted*<br>p<0.05: 0/10	*Unweighted*<br>p<0.05: 0/10<br>*Weighted*<br>p<0.05: 0/10(1)
