# Supplementary material for: Resource redistribution in polydomous ant nest networks: local or global?
Source: Behav Ecol. 2014 Jun 30;25(5):1183–91. doi: 10.1093/beheco/aru108 (PMC4160112; doi:10.1093/beheco/aru108)
Supplement: Supplementary Data [file supp_aru108_Supplementary_Data_4.pdf]

Supplementary Data 4: The relationship between the strength of a trail and the trail betweeness. All statistics are Spearmans Rank Correlation tests with a p value constructed from constrained randomisation (see methods). If nest number was less than 10 the statistics were considered unreliable and not included.

**Colony**	**ρ**	**p**
**1**	-0.30	0.09
**2**	-0.19	0.262
**3**	0.009	0.466
**4**	-0.006	0.478
**5**	-0.13	0.313
**6**	-	-
**7**	-0.05	0.418
**8**	-	-
**9**	0.11	0.632
**10**	-0.02	0.468
